# Comprehensive Virome Analysis of Commercial Lilies in South Korea by RT-PCR, High-Throughput Sequencing, and Phylogenetic Analyses

**DOI:** 10.3390/ijms26199598

**Published:** 2025-10-01

**Authors:** Dongjoo Min, Yeonhwa Jo, Jisoo Park, Gyeong Geun Min, Jin-Sung Hong, Won Kyong Cho

**Affiliations:** 1 Interdisciplinary Program in Smart Agriculture, Kangwon National University, Chuncheon 24341, Republic of Korea; perdues2@naver.com (D.M.); pjsbob12@naver.com (J.P.); mean308@kangwon.ac.kr (G.G.M.); 2 Department of Plant Protection and Quarantine, Jeonbuk National University, Jeonju 54896, Republic of Korea; yeonhwajo@gmail.com; 3 Agriculture and Life Sciences Research Institute, Kangwon National University, Chuncheon 24341, Republic of Korea; 4 Department of Plant Medicine, Division of Bioresource Sciences, Kangwon National University, Chuncheon 24341, Republic of Korea

**Keywords:** lilies, plant viruses, RT-PCR, high-throughput sequencing, transcriptome analysis

## Abstract

Viral diseases pose a significant threat to lily (*Lilium* spp.) cultivation; however, large-scale assessments of virus prevalence and diversity in South Korea are limited. This study combined RT-PCR surveys, high-throughput sequencing (HTS), and analyses of 48 lily hybrid transcriptomes to characterize the lily virome. RT-PCR screening of 100 samples from 13 regions showed that 87% were infected, primarily with lily mottle virus (LMoV, 65%), Plantago asiatica mosaic virus (PlAMV, 34%), cucumber mosaic virus (CMV, 34%), and lily symptomless virus (LSV, 25%). Mixed infections were approximately twice as frequent as single infections and were associated with greater symptom severity, particularly in triple-virus combinations. High-throughput sequencing expanded detection to six viruses, including milk vetch dwarf virus (MDV) and lily virus B (LVB), the latter confirmed as a variant of strawberry latent ringspot virus (SLRSV). Near-complete genomes of several viruses were assembled and validated through RT-PCR. Transcriptome mining identified eight virus species across 26 cultivars; PlAMV was the most common, and viral loads varied significantly among hybrids. Phylogenetic analyses revealed close relationships between Korean and Chinese isolates and host-related clustering in PlAMV. These findings highlight the complexity of lily viromes in South Korea and provide essential resources for diagnostics, disease management, and biosecurity.

## 1. Introduction

Lilies (*Lilium* spp.) are popular ornamental plants cultivated worldwide and are important flowers in Korea, alongside roses and chrysanthemums [1,2]. The main types of lilies grown in Korea are Asiatic, longiflorum, and oriental hybrids, each with unique colors and forms. Lilies are primarily used in gardens and floral arrangements, and the roots of some species have traditional medicinal uses [3]. Similar to tulips and daffodils, lilies grow from bulbs and are propagated by planting bulb scales, which require several years to develop into flowering plants [4].

As a consequence of the vegetative propagation of major lily cultivars, lily plants are often infected by a wide range of pathogens, including viruses [5,6]. During propagation, frequent contact with soil or other sources of infection increases the risk of virus transmission. Therefore, it is the most important step to ensure virus-free bulbs [7]. Several viruses have been in lilies worldwide. Among them, lily mottle virus (LMoV), lily symptomless virus (LSV), Plantago asiatica mosaic virus (PlAMV), and cucumber mosaic virus (CMV) are the most common, and are frequently found in major lily cultivars across different countries [5,8,9,10,11]. These viruses can cause various symptoms, reduce plant quality, and significantly lower the commercial value of lilies. Additionally, three other important viruses that frequently infect lilies are strawberry latent ringspot virus (SLRSV), lily virus X (LVX), and Arabis mosaic virus (ArMV) [12,13,14]. These viruses have been reported in various regions and can cause significant symptoms that reduce the ornamental and commercial value of lilies.

Several studies have been conducted worldwide to identify viruses infecting lilies, using techniques such as enzyme-linked immunosorbent assay (ELISA), polymerase chain reaction (PCR), real-time PCR (quantitative PCR), high-throughput sequencing (HTS), and lateral flow immunoassay (LFA) [15,16,17,18,19,20]. Each detection method has its own advantages and limitations. For instance, ELISA and PCR are widely used to detect known viruses because they are simple and cost-effective. In contrast, HTS, when combined with bioinformatics analysis, is a powerful tool for discovering novel viruses without prior information regarding the target, making it especially useful for comprehensive virus identification [21]. Several studies on lily viruses have been conducted in countries with significant lily cultivation, such as the Netherlands, Japan, China, and the United States [8,9,11,22,23]. These studies have helped identify the types and distribution of viruses affecting lilies in those regions. However, comprehensive studies on the prevalence and impact of these viruses in Korean lily cultivars are limited. Therefore, understanding the infection status and viral composition of lilies produced in Korea is essential for developing effective disease management strategies and ensuring the health and quality of domestic lily crops.

To address this gap in the literature, this study investigated virus infections in 100 lily samples collected from 13 geographical regions across South Korea using reverse transcription PCR (RT-PCR). We also analyzed 10 imported lily cultivars, six major cultivation regions, and three different tissues (leaves, flowers, and bulbs) using both HTS and RT-PCR. Additionally, we identified viruses from the transcriptomes of 48 lily hybrid cultivars from a previous study through in silico analysis. Together, these results provide a comprehensive overview of the virome present in commercial lilies.

## 2. Results

### 2.1. Detection and Geographic Distribution of Major Lily Viruses in South Korea Using RT-PCR

**Figure 1:**
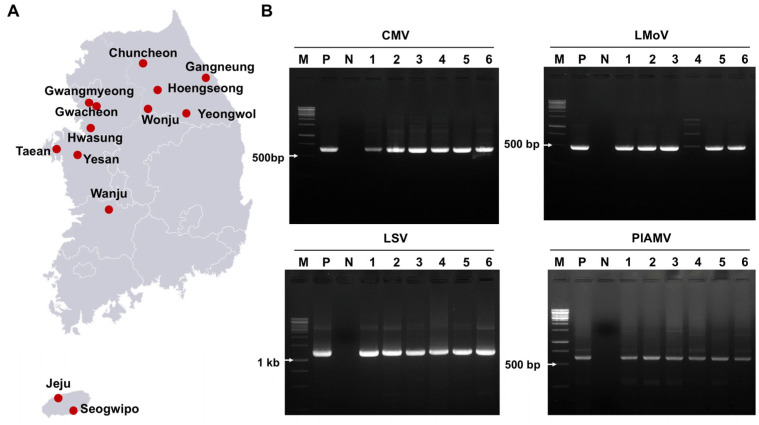
Virus detection in 100 lily samples from South Korea using RT-PCR. (**A**) Map of the 13 regions where lily leaves were collected for virus detection. (**B**) Agarose gel images of RT-PCR products detecting four viruses: CMV, LMoV, LSV, and PlAMV. M = DNA ladder, P = positive control, N = negative control, lanes 1–6 = individual lily samples. Complete RT-PCR results for virus detection are provided in Table S2.

To investigate viral diseases in lilies cultivated in South Korea, 100 leaf samples (both symptomatic and asymptomatic) were collected from 13 major production regions (Figure 1A). RT-PCR screening was conducted for 17 known lily-infecting viruses (Table S1). Overall, 87% of samples tested positive, mainly for CMV, LMoV, LSV, and PlAMV, either as single or mixed infections (Table S2, Figure 1B). Thirteen other viruses, including tomato spotted wilt virus (TSWV), broad bean wilt virus 2 (BBWV2), and lily virus X (LVX), were not detected. Among the infected samples, 30% carried single infections, and 57% showed coinfections.

**Figure 2:**
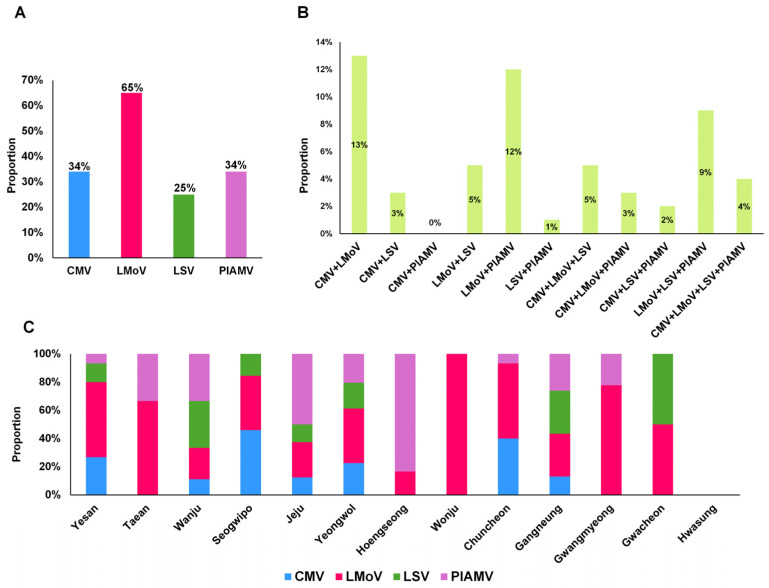
Proportion of virus infections in lily samples and regions of South Korea. (**A**) Proportion of samples positive for the four major lily viruses. (**B**) Proportion of lily samples co-infected with multiple viruses. (**C**) Proportion of virus infections in each geographical region.

LMoV was the most prevalent virus (65%), followed by PlAMV and CMV (34% each), and LSV (25%) (Figure 2A). Coinfections were frequent: CMV + LMoV (13%), LMoV + PlAMV (12%), and LMoV + LSV + PlAMV (9%) were the most common (Figure 2B). Other combinations, such as CMV + PlAMV, LSV + PlAMV, and CMV + LSV + PlAMV, occurred at less than 3%.

Regional variation was also observed (Figure 2C). With the exception of Hwasung, where no viruses were detected, and Wonju, where only LMoV was identified, most regions showed multiple virus infections. Five regions (Yesan, Wanju, Jeju, Yeongwol, and Gangneung) harbored all four major viruses. Seogwipo and Chuncheon had three virus types, whereas Taean, Hoengseong, Gwangmyeong, and Gwacheon showed at least two.

**Figure 3:**
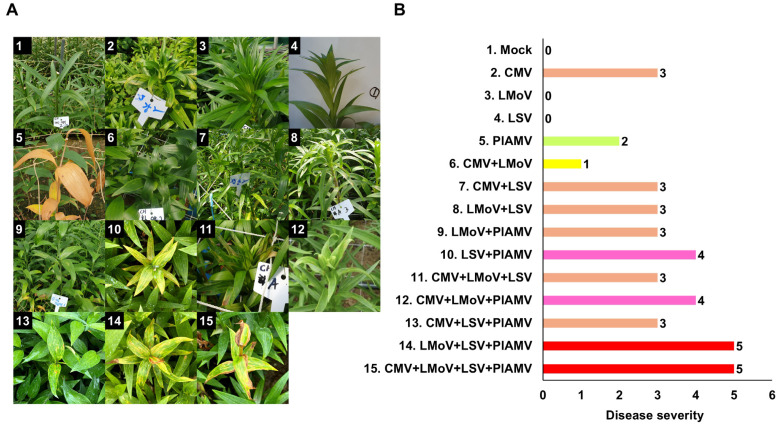
Representative symptoms of viral diseases in lilies caused by single or mixed virus infections. (**A**) Photographs showing diverse virus-induced symptoms under different infection combinations. Each image represents characteristic disease symptoms associated with specific viral infections. The labels containing non-English terms in the figure were used to identify the samples. (**B**) Symptom severity for different virus infections. Numbers indicate the symptom index: 0, symptomless; 1, mosaic; 2, necrosis; 3, necrotic spot; 4, necrotic spot and stripe; 5, necrosis, mosaic, and chlorosis. The different colors indicate the number of coinfected viruses.

A wide range of disease symptoms was observed in lilies, with common signs including leaf mosaics, necrotic spots, and stripes. Viral infections primarily affected leaves and flowers, causing substantial damage to lily quality (Figure 3). RT-PCR results revealed that mixed infections were nearly twice as frequent as single infections, prompting an assessment of symptom severity in relation to infection type (Figure 3A,B). Single infections produced relatively mild symptoms. LMoV and LSV frequently caused no visible symptoms, CMV induced mosaic or necrotic spots (severity 3), and PlAMV caused leaf necrosis (severity 2). Mixed infections, however, resulted in stronger symptoms (Figure 3B). Double infections such as CMV + LSV, LMoV + LSV, and LMoV + PlAMV caused severity 3, whereas LSV + PlAMV reached severity 4. Triple infections presented variable outcomes: CMV + LMoV + LSV and CMV + LSV + PlAMV showed severity 3, CMV + LMoV + PlAMV reached severity 4, and LMoV + LSV + PlAMV produced the highest severity at 5.

### 2.2. Identification of Plant Viruses Infecting Lilies in South Korea by HTS

HTS was performed to identify viruses infecting lilies in South Korea. Samples were collected from multiple cultivars and tissues (bulbs, leaves, flowers), with some pooled for sequencing (Table 1). Total RNA was extracted from 20 samples, and eight libraries were prepared for HTS. Sequencing data were deposited in the NCBI SRA database with corresponding accession numbers.

HTS yielded 18,175,058 viral reads and 73 viral contigs corresponding to six virus species (Table 2). Four major viruses (LSV, LMoV, PlAMV, CMV) and two additional viruses (milk vetch dwarf virus (MDV) and lily virus B (LVB)) were identified. CMV and LSV were detected only in leaf (LL) and bulb (LB) libraries. LMoV was detected in five libraries (LB, LL, LFL, LFLS, LFLY), while PlAMV was found in six (LL1, LL2, LL3, LFL, LFLS, LFLY). MDV contigs (15 total) were identified exclusively in LFLS, and LVB contigs (3 total) only in LFLY. For LB and LL, total RNA was extracted from the same plant. Viral reads from bulbs were over four times higher than those from leaves, and flowers harbored greater viral diversity than leaves.

Nearly complete viral genomes were reconstructed for several isolates, including LSV (LL, LB), LMoV (LL, LFL, LFLS), CMV (LL, LB; three RNA segments each), PlAMV (LL3, LFL), and LVB (LFLY; two RNA segments) (Table S3). Partial MDV DNA segments (U1, N, U2, M, S, U4) were also obtained.

**Figure 4:**
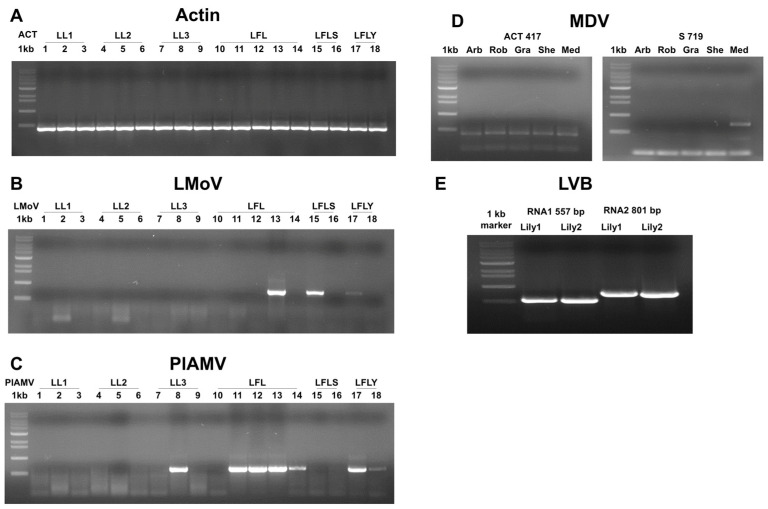
Confirmation of HTS results by RT-PCR using virus-specific primers. (**A**) The lily *actin gene* was used as a positive control to verify total RNA quality. (**B**) RT-PCR using LMoV-specific primers showed amplification in samples 13, 15, and 17. (**C**) RT-PCR using PlAMV-specific primers showed amplification in samples 8, 11, 12, 13, 14, 17, and 18. (**D**) RT-PCR using MDV S segment-specific primers indicated amplification in the Medusa (Med) sample among different cultivars. (**E**) RT-PCR using LVB RNA1- and RNA2-specific primers showed amplification in two Yelloween plants.

RT-PCR using virus-specific primers confirmed the HTS results (Figure 4). The lily actin gene was used as a positive control (Figure 4A). LMoV was confirmed in cultivars Sheila (sample 13), Siberia (15), and Yelloween (17) (Figure 4B). PlAMV was confirmed in seven samples (Figure 4C). The MDV S segment was detected in the Medusa cultivar (flower tissue) (Figure 4D), and LVB RNA1 and RNA2 were amplified only from Yelloween (Figure 4E).

### 2.3. Identification of Viral Sequences from 48 Lily Hybrid Transcriptomes

To expand our understanding of viruses infecting lilies, we analyzed transcriptome data from 48 lily hybrid cultivars, many of which are commercially available, based on a previous study [24]. A total of 3125 viral contigs were identified, corresponding to eight virus species: lily virus A (LVA), Cycas necrotic stunt virus (CNSV; two RNA segments), PlAMV, LSV, LMoV, SLRSV (two RNA segments), lily amalgavirus 1 (LAV1), and lily amalgavirus 2 (LAV2) (Table 3).

**Figure 5:**
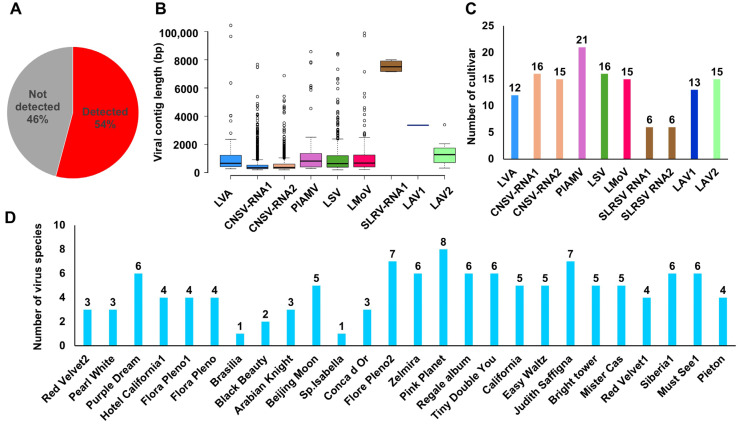
Virus detection patterns in 48 lily hybrid transcriptomes. (**A**) Proportion of virus detection in 48 lily hybrid transcriptomes. (**B**) Box plots showing viral contig distribution for each virus. Each viral RNA segment is indicated by a different color. (**C**) Number of lily hybrid cultivars in which each virus was identified. Each viral RNA segment is indicated by a different color. (**D**) Number of virus species identified per lily hybrid cultivar.

Mapping raw transcriptome reads to reference genomes revealed viral reads, coverage, and fragments per kilobase of transcript per million mapped reads (FPKM) values (Table S4). Viral sequences were detected in 26 of 48 transcriptomes (Figure 5A). Several contigs covered nearly complete genomes (Figure 5B). CNSV contigs were the most abundant (1644 for RNA1, 728 for RNA2), and fewer contigs were observed for other viruses (e.g., four for SLRSV RNA1, one for LAV1, and 11 for LAV2). Overall, 25 nearly complete RNA fragments were obtained across the eight virus species.

Virus infection frequency varied by species: PlAMV was the most common (21 cultivars), followed by CNSV (RNA1 in 16, RNA2 in 15), LSV (16), LMoV (15), and LAV2 (15). LVA and LAV1 were found in 12 and 13 cultivars, respectively, whereas SLRSV appeared in only six (Figure 5C). Most cultivars harbored more than two viruses, except Brasilia and Sp. Isabella. Pink Planet carried the highest number (8 species), followed by Flore Pleno2 and Judith Saffigna (7 each) (Figure 5D).

**Figure 6:**
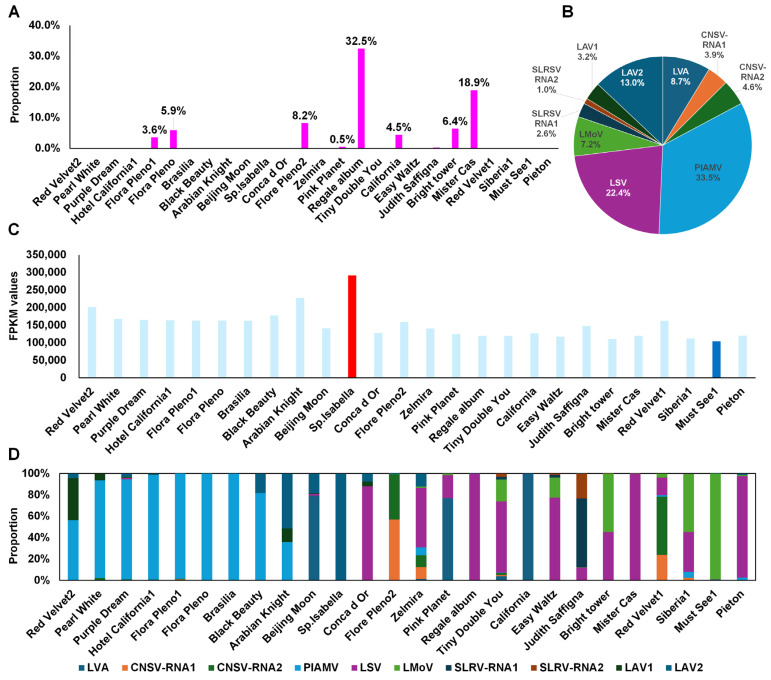
Proportion and quantification of viruses in lily transcriptome data. (**A**) Proportion of viral reads compared to total transcriptome reads. (**B**) Pie chart showing the relative abundance of individual identified viruses by FPKM. (**C**) FPKM values of viruses in each lily transcriptome. The red and blue bars indicate the highest and lowest values. (**D**) Viral composition per lily transcriptome.

The proportion of viral reads relative to total reads was generally low, close to 0% (Figure 6A). Exceptions included Regale album (32.5%), Mister Cas (18.9%), and Flore Pleno2 (8.2%). Based on combined FPKM values, PlAMV was the dominant virus (33.5%), followed by LSV (22.4%), LAV2 (13%), and LVA (8.7%) (Figure 6B). Among individual transcriptomes, Sp. Isabella had the highest viral FPKM, while Must See1 had the lowest (Figure 6C).

Virus dominance also varied by cultivar (Figure 6D). PlAMV was abundant in Hotel California1, Flore Pleno1, Flore Pleno, and Brasilia. LAV2 dominated in Arabian Knight and Sp. Isabella. LSV was abundant in Conca d’Or, Regale album, Tiny Double You, Easy Waltz, Mister Cas, and Peton. LVA dominated in California and Beijing Moon. LMoV was enriched in Must See1, Siberia1, and Bright Tower.

### 2.4. Phylogenetic Analyses Based on Assembled Viral Genomes

**Figure 7:**
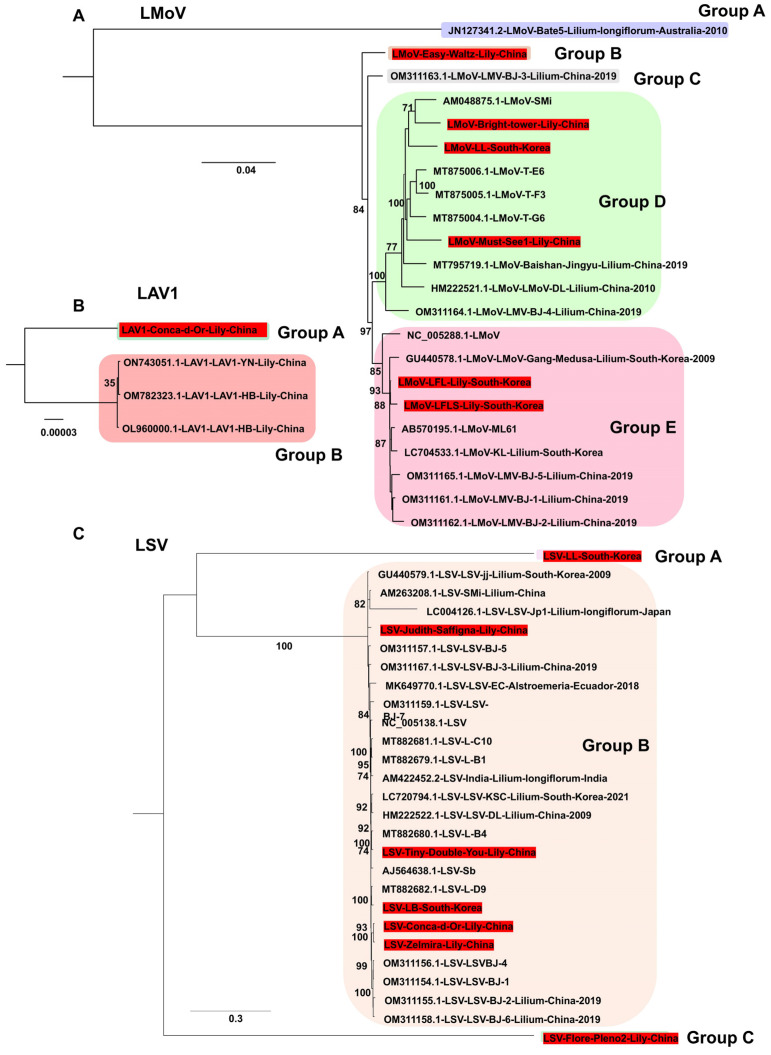
Phylogenetic relationships of LMoV, LAV1, and LSV genomes obtained in this study compared with known viral genomes. (**A**) Phylogenetic tree of 22 LMoV isolates, including six genomes generated in this study. The tree was constructed using the best-fit substitution model identified by the Bayesian information criterion (BIC): GTR + F + I + G4. (**B**) Phylogenetic tree of four LAV1 genomes, including one newly sequenced genome from this study. The best-fit model selected by BIC was F81 + F. (**C**) Phylogenetic tree of 27 LSV genomes, including five newly identified genomes from this study. The SYM + G4 model was determined as the best fit by BIC for tree construction. Each group is indicated by a different color. The red box represents the viral genome derived from this study.

Phylogenetic analyses were performed using nearly complete viral genomes obtained in this study, together with reference genomes. For LMoV, six genomes were obtained (three from South Korea, three from China). Analysis of 22 LMoV genomes revealed five groups (Figure 7A). Group A, which contained an Australian isolate (Bate5), was distantly related to the other groups. Most isolates were clustered in groups D and E (10 isolates each). Among the Korean isolates, LMoV-LL belonged to group D, and LMoV-LFL and LMoV-LFLS were placed in group E. For LAV1, only four genomes are available, all from China (Figure 7B). The newly identified genome from this study clustered separately, showing genetic divergence from the other three isolates. For LSV, seven genomes were obtained (two from South Korea and five from China). Based on 27 LSV genomes, three groups were identified (Figure 7C). The Korean isolate LSV-LL and the Chinese isolate LSV-Flore Pleno2 (Group A) were distantly related to the 25 other isolates, which clustered closely in Group B.

**Figure 8:**
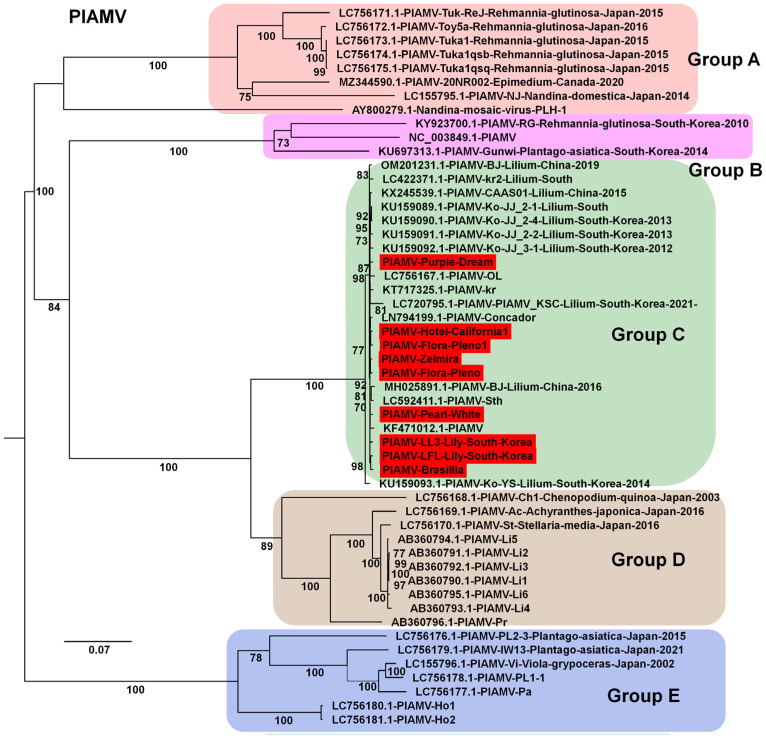
Phylogenetic relationships of PlAMV genomes obtained in this study compared with known isolates. Phylogenetic tree of 52 PlAMV isolates, including nine new genomes identified in this study. The tree was constructed using the best-fit substitution model determined by Bayesian information criterion (BIC): GTR + F + I + G4. Each group is indicated by a different color. The red box represents the viral genome derived from this study.

For PlAMV, nine genomes were obtained (two from South Korea and seven from China). Phylogenetic analysis of 52 genomes showed clear divergence into several groups (Figure 8). All isolates from this study clustered in Group C, along with other lily-derived isolates. Group A consisted mainly of isolates from *Rehmannia glutinosa* in Japan.

**Figure 9:**
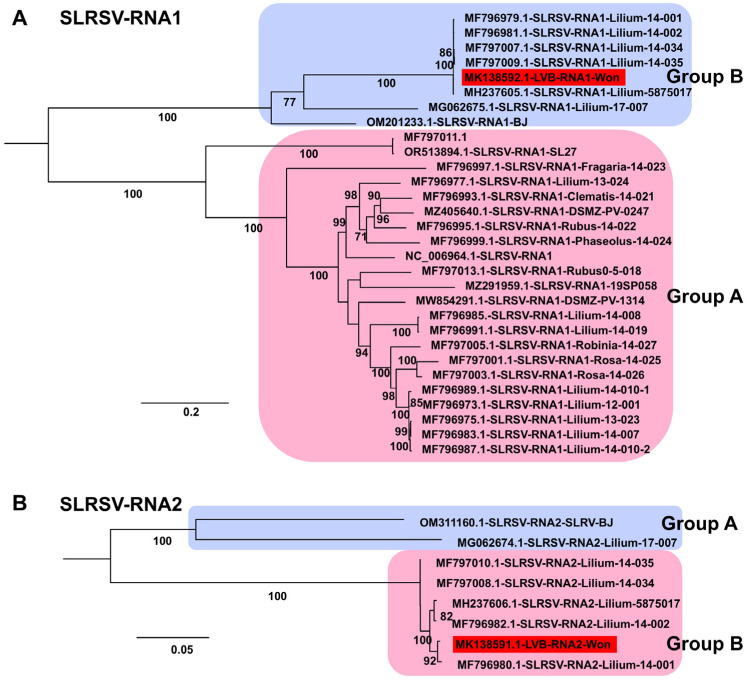
Phylogenetic relationships of SLRSV genomes obtained in this study compared with known isolates. (**A**) Phylogenetic tree of 30 SLRSV RNA1 isolates, including one novel genome identified in this study. The tree was constructed using the best-fit substitution model identified by the Bayesian information criterion (BIC): GTR + F + I + G4. (**B**) Phylogenetic tree of eight SLRSV RNA2 isolates, including one newly identified genome from this study. The best-fit substitution model determined by BIC was TIM2 + F + I. Each group is indicated by a different color. The red box represents the viral genome derived from this study.

LVB, identified from Lilium cv. Yelloween in 2018, was determined not to be novel under the International Committee on Taxonomy of Viruses (ICTV) criteria for the genus Stralarivirus. Phylogenetic analyses demonstrated that LVB is a member of SLRSV, genus Stralarivirus, family Secoviridae (Figure 9A). In analyses of both SLRSV RNA1 (50 sequences) and RNA2 (8 sequences), the LVB-Won isolate clustered with SLRSV members in Group B.

**Figure 10:**
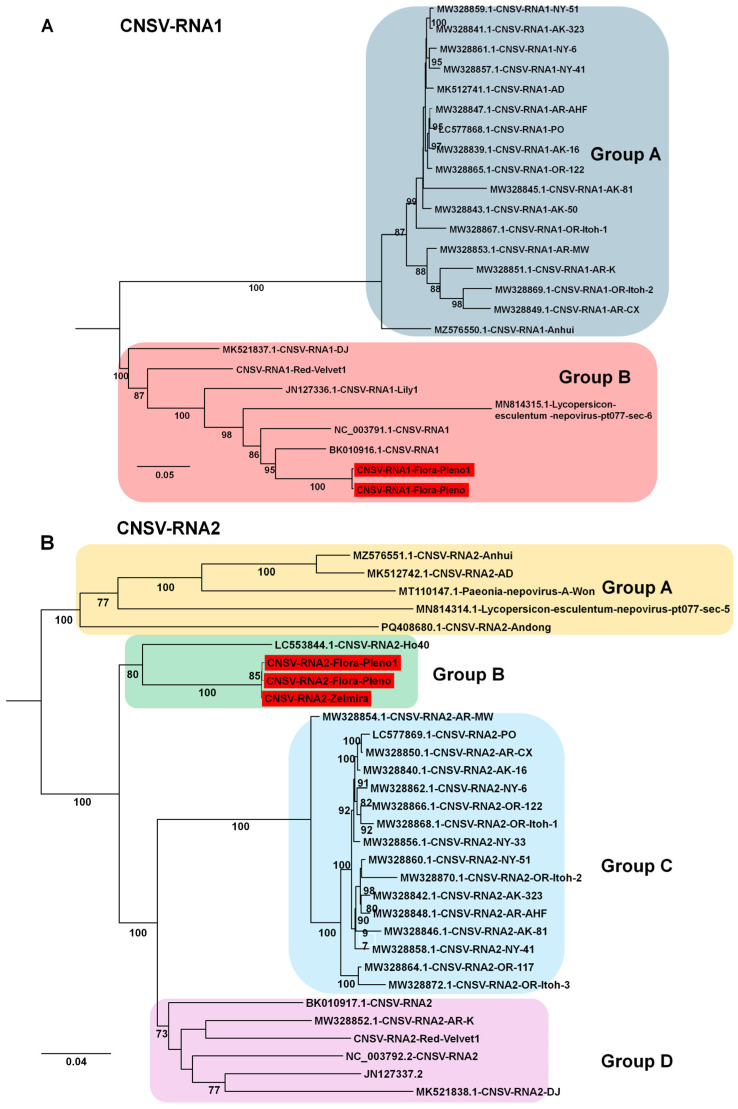
Phylogenetic relationships of CNSV genomes obtained in this study compared with known isolates. (**A**) Phylogenetic tree of 25 CNSV RNA1 isolates, including two novel genomes identified in this study. The tree was constructed using the best-fit substitution model identified by the Bayesian information criterion (BIC): TIM2 + F+I + G4. (**B**) Phylogenetic tree of 31 CNSV RNA2 isolates, including three newly identified genomes from this study. The best-fit substitution model determined by BIC was TVM + F + I + G4. Each group is indicated by a different color. The red box represents the viral genome derived from this study.

For CNSV, two RNA1 genomes and three RNA2 genomes were obtained from Chinese isolates. Phylogenetic analysis of 25 RNA1 sequences revealed two groups: Group A (17 isolates) and Group B (8 isolates, including the two Chinese isolates from this study) (Figure 10A). Analysis of 31 RNA2 sequences revealed four groups. All three isolates obtained here clustered in Group B, while Group C contained the largest number of isolates (16). In addition, two CMV genomes (isolates LL and LB) and two LVA genomes (isolates Beijing Moon and California) were obtained. The CMV genomes were closely related to previously reported Korean lily isolates, whereas the two newly identified LVA genomes represent two of only three currently available for this virus.

## 3. Discussion

This study provides a comprehensive overview of viruses infecting lilies in South Korea through an integrated approach combining RT-PCR surveys, high-throughput sequencing (HTS), and reanalysis of hybrid lily transcriptomes from China based on a previous study [24]. Using these complementary methods, we characterized the prevalence and diversity of major viruses, their coinfection patterns, geographic distribution, and phylogenetic relationships in a global context.

The RT-PCR survey of 100 samples from 14 production regions revealed widespread viral infections, with 87% of samples testing positive for at least one virus. Four viruses, LMoV, PlAMV, CMV, and LSV, dominated the virome, consistent with previous studies conducted in Korea [15]. LMoV was the most prevalent (65%), followed by PlAMV and CMV (34% each) and LSV (25%). These findings confirm LMoV as the leading viral threat to lilies in South Korea and align with reports from other major lily-growing countries [8,10,11]. Our study found that mixed virus infections in lilies were more common than single infections. Some combinations, such as LMoV + PlAMV and CMV + LMoV occurred frequently. In single virus infections, LMoV and LSV often showed no symptoms, whereas CMV and PlAMV caused leaf mottling or damage. Mixed infections induced more severe symptoms, especially when three viruses (LMoV, LSV, and PlAMV) were present together. These results show that viral interactions cause an increase in disease severity, making it harder to diagnose and manage infections. As described previously, mixed infections of plant viruses can result in synergistic or antagonistic interactions that influence viral evolution, pathogenesis, and epidemiology, making them critical to understanding plant disease development and control strategies [25,26]. A previous study on lilies from Jeju Island also showed that mixed infections were more common than single ones, with PlAMV and LSV appearing most frequently in mixed infections [27]. The study showed that LSV alone did not cause symptoms; however, when mixed in combination with other viruses such as LMoV or CMV, plants developed leaf deformities and spots. PlAMV caused severe leaf damage, especially in mixed infections, which reduced the quality and market value of lilies. Both studies agree that mixed infections involving these viruses are widespread and cause more severe symptoms than single virus infections. This highlights the need for careful monitoring and control strategies that consider multiple viruses to protect lily crops. Therefore, producing virus-free plants through tissue culture methods, such as shoot meristem tip culture and in vitro thermotherapy, is essential [28]. Geographic patterns of virus incidence further highlight this complexity. While most regions showed multiple virus infections, some showed unique profiles, such as Wonju, where only LMoV was present, and Hwasung, where no viruses were detected. Notably, CMV was detected only in Gangwon Province, suggesting a region-specific infection pattern of certain viruses in lilies. In contrast, regions such as Yesan, Wanju, Jeju, Yeongwol, and Gangneung exhibited all four major viruses simultaneously, acting as centers of high viral diversity. These regional differences suggest that localized monitoring is essential for identifying hotspots of infection and preventing further dissemination through bulbs and cut flowers, which are key export products in South Korea.

HTS analyses have broadened the known range of viruses infecting lilies and allowed the assembly of near-complete viral genomes, similar to findings in other bulb-propagated plants such as garlic and Chinese narcissus [5,29,30]. Along with confirming the four major viruses identified by RT-PCR, we also detected MDV and LVB. We previously reported MDV infection in both lily and pepper plants [31,32]. Although not traditionally considered lily pathogens, the availability of MDV genomic fragments provides a resource for future monitoring. LVB was identified as a variant of SLRSV, placing it within the genus *Stralarivirus*. This classification emphasizes the importance of robust phylogenetic and taxonomic frameworks in virus identification. Notably, both MDV and LVB were identified in lily flower tissues with relatively high levels of viral abundance. As previously demonstrated, selecting the appropriate plant tissues is very important for virus identification, rather than solely using leaves as the typical plant material [29,33]. Moreover, HTS enabled the recovery of complete or nearly complete genomes for several viruses, including multiple isolates of CMV, LMoV, LSV, PlAMV, and LVB. These data provide essential resources for evolutionary studies, diagnostic development, and comparative virology. Notably, targeted RT-PCR validation in cultivars such as Sheila, Siberia, Yelloween, and Medusa confirmed the reliability of the HTS-based findings. Due to funding limitations, some samples in our study were pooled; however, the RT-PCR approach successfully identified the target virus infections, highlighting the necessity of using multiple methods for comprehensive virome studies [34].

The reanalysis of 48 publicly available lily hybrid transcriptomes expanded our understanding of lily viromes at the cultivar level. A total of eight virus species were detected: LVA, CNSV, PlAMV, LSV, LMoV, SLRSV, LAV1, and LAV2. Viruses occurred in 26 transcriptomes, indicating that more than half of the hybrids tested carried viral sequences. PlAMV was the most prevalent species, followed by CNSV, LSV, LMoV, and LAV2. Some cultivars exhibited particularly high viral diversity, with Pink Planet containing eight viruses and Flore Pleno2 and Judith Saffigna each containing seven, suggesting that these hybrids may act as reservoirs for multiple viral species. In contrast, Brasilia and Sp. Isabella carried only one or two viruses, reflecting variation in cultivar susceptibility. Analysis of viral proportions within transcriptomes generally showed low levels but revealed high accumulation in some cultivars, including Regale album (32.5%), Mister Cas (18.9%), and Flore Pleno2 (8.2%). The dominant virus also varied by cultivar, demonstrating the cultivar-specific nature of viral prevalence in lilies. These results align with previous studies exploring viral diversity through transcriptome reanalysis and show that some cultivars can act as reservoirs for multiple viruses, while others are less susceptible [5,35,36]. Notably, PlAMV, LSV, and LMoV were the dominant viruses detected in multiple cultivars. This finding is consistent with their broad host ranges and high prevalence reported in the recent literature across East Asia and globally. These viruses were found in diverse lily hybrids, with cultivars such as Pink Planet and Flore Pleno2 harboring up to eight virus species, whereas others, such as Brasilia, carried only a few. This distribution pattern highlights differential cultivar susceptibility and the potential role of certain cultivars as virus reservoirs. PlAMV, in particular, is significant due to its widespread geographic distribution, high infection rates in ornamental lilies, and ability to spread through soil and bulb trade. These characteristics contribute significantly to its epidemiological impact. Our findings support previous reports that PlAMV is globally widespread and consistently present across many commercial lily cultivars, underscoring its role as a key reservoir virus. Similarly, LSV and LMoV showed consistent prevalence patterns across cultivars, reinforcing their importance in lily viromes given their known effects on plant health and commercial value.

Phylogenetic analyses of assembled genomes placed lily viruses within a broader global context. For LMoV, analysis of 22 genomes identified five groups, with Korean isolates clustering mainly in Groups D and E in close relation to Chinese isolates, whereas an Australian isolate represented a divergent lineage. For LAV1, only four genomes were available, all from China, and the newly identified isolate in this study was genetically distinct, highlighting intraspecific variation but also underscoring the limited available data. For LSV, most isolates grouped within Group B, whereas the Korean LSV-LL and a Chinese isolate clustered separately in Group A, indicating divergence within the species. PlAMV analysis revealed strong host associations, with all lily-derived isolates, including those from this study, clustering in Group C, whereas isolates from *Rehmannia glutinosa* in Japan clustered in Group A. LVB was consistently placed within SLRSV Group B in both RNA1- and RNA2-based trees, confirming its taxonomic assignment to the genus *Stralarivirus*. CNSV exhibited lineage diversity, with isolates from this study clustering in Group B for both RNA1 and RNA2, while multiple groups were defined overall, reflecting genetic variability within the species. Along with previous studies, our phylogenetic analyses across diverse viral genomes clarify evolutionary relationships, regional diversity, and interspecies variation among lily viruses [11,37,38].

Together, these findings illustrate the virome complexity of lilies in South Korea, the high prevalence of mixed infections, and the strong connections between Korean viral populations and those in other countries, especially China. They also highlight the complementarity of RT-PCR, HTS, and transcriptome analyses in identifying both dominant and previously underrecognized viruses.

## 4. Materials and Methods

### 4.1. Sample Collection for Disease Symptoms and Lily Virus Detection Using RT-PCR

Field sampling of lilies was conducted in major cultivation regions across South Korea from April to June 2020 to investigate viral infections. Samples were collected from five provinces, including Gwangmyeong, Gwacheon, and Hwasung in Gyeonggi-do; Yesan and Taean in Chungcheongnam-do; Wanju in Jeonbuk-do; Seogwipo and Jeju in Jeju-do; and Yeongwol, Hoengseong, Wonju, Chuncheon, and Gangneung in Gangwon-do. Leaf samples were taken from plants showing virus-like symptoms such as mosaic patterns, streaks, or leaf deformation, and asymptomatic plants were also collected as controls. Samples were placed in sterile plastic bags, kept on ice, and transported to the laboratory for analysis. Visual inspections in the field were conducted to record symptom type and severity of symptoms, and representative photographs were taken at each site. Symptom severity was evaluated using a numerical index: 0, symptomless; 1, mosaic; 2, necrosis; 3, necrotic spot; 4, necrotic spot and stripe; and 5, necrosis, mosaic, and chlorosis.

### 4.2. Sample Collection for Lily Virus Identification Using HTS

To identify viruses infecting lilies (*Lilium* spp.), plant samples were collected from both imported cultivars and domestically cultivated varieties. A total of 30 different lily bulb cultivars imported into Korea were purchased and grown under controlled greenhouse conditions. After planting, the lilies were carefully monitored for virus-related symptoms in 2018. Virus-like symptoms, such as leaf mottling, streaking, or deformation, were recorded during the growth period. Most imported cultivars did not exhibit distinct symptoms typically associated with viral infections. Based on preliminary observations, 10 cultivars (Casablanca, Sorbonne, Black Beauty, Kensington, Amarossi, Zambesi, Cadenza, Donato, Myth, Arbataxout) were selected for HTS library preparation.

Field surveys were also conducted in five major domestic lily cultivation regions: Suwon (Robina cultivar), Chilgok (Gracia), Iksan (Sheila, Medusa, Siberia cultivars), Jeju (Siberia), and Seosan (Yelloween). Leaf samples were collected from randomly selected plants exhibiting potential virus symptoms or abnormal growth patterns. Representative samples from each location were transported to the laboratory for further analysis. Moreover, leaf and bulb samples showing viral symptoms were collected from an unknown lily cultivar grown in Chuncheon to compare viromes between leaf and bulb tissues.

For HTS, lily virome samples were prepared using a systematic RNA pooling strategy. For leaf libraries (LL1, LL2, LL3), total RNA from three individual cultivars grown in the same greenhouse was extracted separately and then combined in equal proportions for each library (Casablanca, Sorbonne, Black Beauty for LL1; Kensington, Amarossi, Zambesi for LL2; and Cadenza, Donato, Myth for LL3). Flower libraries followed a similar method: LFL pooled RNA from flowers of five cultivars across diverse regions, while LFLS and LFLY each combined RNA from Siberia and Yelloween cultivars collected at multiple locations, respectively. For the bulb and leaf samples from Chuncheon, RNA was prepared individually without pooling. This strategy allowed each HTS library to represent either three samples per leaf pool, five per primary flower pool, two per specialized cultivar-tissue pool, or one individual sample, optimizing both detection sensitivity and clarity of provenance for virome characterization.

### 4.3. Total RNA Extraction from Collected Lily Samples

Leaf samples were rapidly frozen in liquid nitrogen immediately after collection and kept at −80 °C until further analysis. Approximately 100 mg of leaf tissue was subjected to total RNA isolation using either the BCSTM Plant RNA Prep Kit (Biocube System Inc., Suwon, Republic of Korea) or the RNeasy Plant Mini Kit (Qiagen, Hilden, Germany), following the respective manufacturer’s recommendations. The concentration and purity of the extracted RNA were measured using a spectrophotometer (NanoDrop; Thermo Fisher Scientific, Waltham, MA, USA), and RNA integrity was assessed by agarose gel electrophoresis. Extracted RNA was stored at −80 °C until use.

### 4.4. Detection of Lily-Infecting Viruses by RT-PCR

RT-PCR assays were performed to screen for sixteen different viruses: CMV, LMoV, LSV, PlAMV, tomato spotted wilt virus (TSWV), broad bean wilt virus 2 (BBWV2), tobacco mosaic virus (TMV), lily virus X (LVX), MDV, apple stem grooving virus (ASGV), tobacco rattle virus (TRV), tomato aspermy virus (TAV), tobacco ringspot virus (TRSV), tomato ringspot virus (ToRSV), SLRSV, shallot yellow stripe virus (SYSV), and narcissus mosaic virus (NMV). Complementary DNA (cDNA) was synthesized from extracted RNA by reverse transcription using M-MLV reverse transcriptase (Promega, Madison, WI, USA) and random hexamer primers (Takara Bio, Shiga, Japan) in a 20 µL reaction system.

To detect and confirm virus infections, complementary DNA (cDNA) was synthesized from total RNA using M-MLV reverse transcriptase (Promega, Madison, WI, USA) with random hexamer primers (Takara Bio, Shiga, Japan) in a 20 µL reaction volume. The reverse transcription reaction was carried out at 42 °C for 60 min, followed by enzyme inactivation at 92 °C for 5 min. PCR amplification of viral target regions was performed using virus-specific primers (listed in Supplementary Table S1) with Supreme Tech PCR reagents (Doctor Bio, Daejeon, Republic of Korea) in a Bio-Rad thermal cycler. Each 20 µL PCR contained 1 µL of cDNA template, 10 pmol of each primer, and other PCR components as per the manufacturer’s instructions. PCR cycling conditions were optimized for each primer pair to maximize specificity and sensitivity. Thermal cycling was performed beginning with a 3 min denaturation at 94 °C, followed by 35 cycles consisting of 94 °C for 30 s, primer-specific annealing at 56–60 °C, and elongation at 72 °C for 20 s to 1 min depending on the target size. A final extension step was carried out at 72 °C for 5 min. Information on annealing temperatures and extension times for each primer pair is provided in Supplementary Table S1. PCR amplicons were separated on 1% agarose gels stained with ethidium bromide and observed under UV light using a Gel-Doc imaging system (Bio-Rad, Richmond, CA, USA). Selected products were purified and sequenced to confirm the presence of the respective viruses.

To confirm the results from pooled samples obtained by HTS, RT-PCR was conducted using the following primer sets: LrACT-F1/LrACT-R1, LMoV-8616F1/LMoV-9437R1, PlAMV-5362F1/PlAMV-5985R1, MDV-S-232F1/MDV-S-950R1, LVB-RNA1-3689F1/LFLY-RNA1-4245R1, and LFLY-RNA2-1482F1/LFLY-RNA2-2282R1.

### 4.5. Comprehensive Methodology for Lily Virome Analysis Using HTS and Bioinformatics Tools

For virome characterization, RNA libraries were constructed with the NEBNext Ultra RNA Library Prep Kit for Illumina (New England Biolabs, Ipswich, MA, USA) according to the supplier’s instructions. The libraries were sequenced as paired-end reads (2 × 101 bp) using the Illumina HiSeq 2000 platform at Macrogen (Seoul, South Korea). Quality control of the raw FASTQ reads was performed using BBDuk version 39.01, a component of the BBMap package, to trim adapters and filter out low-quality sequences (https://archive.jgi.doe.gov/data-and-tools/software-tools/bbtools/bb-tools-user-guide/bbduk-guide/) accessed on 1 June 2025. The cleaning process included adapter removal, quality trimming from both ends at a threshold of Q15, and discarding reads shorter than 36 base pairs or containing more than one ambiguous base. Cleaned reads were assembled de novo using both Trinity v2.15.0 and MEGAHIT v1.2.9 under default configurations [39,40]. Assembled contigs were analyzed using a BLASTX against the NCBI viral protein database with an E-value cutoff of 1 × 10^−10^ to identify viral-related sequences. Viral genomes assembled from pooled HTS data were validated by targeted RT-PCR in individual cultivars. When multiple viral variants or strains were present, only contigs with high read support and consistency with known reference sequences were considered nearly complete genomes.

Viral contigs were further confirmed by BLASTX against the NCBI non-redundant protein database and classified by virus species. For quantitative analysis, raw reads were aligned to reference viral genomes using BBMap with default settings (https://archive.jgi.doe.gov/data-and-tools/software-tools/bbtools/bb-tools-user-guide/bbmap-guide/) accessed on 1 June 2025. Viral read coverage and transcript abundance, expressed as fragments per kilobase of exon per million mapped fragments (FPKM), were calculated from the alignment files. Viral genomes were annotated by identifying complete open reading frames (ORFs) using the ORFfinder tool from NCBI (https://www.ncbi.nlm.nih.gov/orffinder/, accessed on 1 June 2025). Reverse complementary sequences were generated as needed using a DNA Reverse Complement Calculator (https://www.qiagen.com/us/applications/enzymes/tools-and-calculators/reverse-complement-converter, accessed on 1 June 2025). Predicted ORFs were further analyzed by BLASTX against the non-redundant protein database to characterize viral genomic features, such as conserved motifs and functional elements, through comparison with known viral genomes. All software and data were accessed as of 1 June 2025. 

For in silico virus identification in Chinese hybrid lilies, we selected 48 lily transcriptomes from BioProject PRJNA1037021 in the SRA database. Each SRA file was downloaded using commands such as: wget ftp://ftp.sra.ebi.ac.uk/vol1/fastq/SRR314/094/SRR31479294/SRR31479294_1.fastq.gz, ftp://ftp.sra.ebi.ac.uk/vol1/fastq/SRR314/094/SRR31479294/SRR31479294_2.fastq.gz. After downloading, the SRA data were converted to FASTQ format using the command: fasterq-dump–split-files SRR31479294. After the cleaning process described above, high-quality reads were assembled de novo using MEGAHIT. Virome analysis then followed the same pipeline as for the HTS data generated in this study. This included viral sequence identification, genome validation, and quantification. This method allowed reliable detection of viruses in publicly available lily transcriptome data.

### 4.6. Phylogenetic Analysis of Viral Sequences Using MAFFT and IQ-TREE

Viral contigs obtained from assemblies were aligned using MAFFT v7 under the auto setting, with terminal untranslated regions trimmed prior to further analysis [41]. The most appropriate nucleotide substitution model for each dataset was determined in advance of tree construction. Maximum likelihood phylogenies were generated with IQ-TREE v2.4.0, with 1000 bootstrap replicates applied to estimate branch reliability [42]. The resulting trees were visualized and formatted in Figtree v1.4.4 (http://tree.bio.ed.ac.uk/software/figtree/, accessed on 1 June 2025).

## 5. Conclusions

By combining RT-PCR surveys, HTS, and transcriptome mining, we provide a comprehensive characterization of the virome infecting lilies in South Korea. Lilies were found to harbor a diverse array of viruses, with high rates of mixed infections and significant variation in infection frequencies across regions and cultivars. HTS enabled the assembly of complete viral genomes and detected additional viruses not identified by RT-PCR. Transcriptome reanalysis broadened the spectrum of identified viruses and demonstrated the cultivar-specific nature of viral prevalence. Phylogenetic analyses clarified relationships and genetic diversity across major lily viruses. Together, these findings provide a solid foundation for future molecular diagnostics, surveillance, and comparative viral genomics in lilies.

## Figures and Tables

**Table 1:** Summary of lily samples used for virome analysis with HTS.

Index	Cultivar	Geographical Region	Tissues	Library Name
1	Casablanca	Green house in Seoul	Leaves	LL1
2	Sorbonne	Green house in Seoul	Leaves	LL1
3	Black beauty	Green house in Seoul	Leaves	LL1
4	Kensington	Green house in Seoul	Leaves	LL2
5	Amarossi	Green house in Seoul	Leaves	LL2
6	Zambesi	Green house in Seoul	Leaves	LL2
7	Cadenza	Green house in Seoul	Leaves	LL3
8	Donato	Green house in Seoul	Leaves	LL3
9	Myth	Green house in Seoul	Leaves	LL3
10	Arbatax	Green house in Seoul	Flowers	LFL
11	Robina	Suwon	Flowers	LFL
12	Gracia	Chilgok	Flowers	LFL
13	Sheila	Iksan	Flowers	LFL
14	Medusa	Iksan	Flowers	LFL
15	Siberia	Iksan	Flowers	LFLS
16	Siberia	Jeju	Flowers	LFLS
17	Yelloween	Iksan	Flowers	LFLY
18	Yelloween	Seosan	Flowers	LFLY
19	Unknown	Chuncheon	Bulbs	LB
20	Unknown	Chuncheon	Leaves	LL

For some samples, total RNA extracted from individual samples was pooled for library preparation. LB = lily bulbs, LL = lily leaves, LFL = lily flowers, LFLS = lily flowers of the Siberia cultivar, and LFLY = lily flowers of the Yelloween cultivar.

**Table 2:** Summary of plant viruses identified in diverse lily cultivars using HTS.

Library Name	Identified Virus	Family	Length	No. of Viral Reads	No. of Viral Contigs
LB	Lily symptomless virus	*Betaflexiviridae*	8394	2,928,217	1
	Lily mottle virus	*Potyviridae*	9644	114,967	16
	Cucumber mosaic virus RNA1	*Bromoviridae*	3357	1,737,976	3
	Cucumber mosaic virus RNA2	*Bromoviridae*	3050	1,157,296	1
	Cucumber mosaic virus RNA3	*Bromoviridae*	2216	5,832,071	1
LL	Lily symptomless virus	*Betaflexiviridae*	8394	131,998	2
	Lily mottle virus	*Potyviridae*	9644	5532	2
	Cucumber mosaic virus RNA1	*Bromoviridae*	3357	1,395,491	3
	Cucumber mosaic virus RNA3	*Bromoviridae*	2216	1,217,009	1
LL1	Plantago asiatica mosaic virus	*Alphaflexiviridae*	6128	9	3
LL2	Plantago asiatica mosaic virus	*Alphaflexiviridae*	6128	22	2
LL3	Plantago asiatica mosaic virus	*Alphaflexiviridae*	6128	2552	1
LFL	Lily mottle virus	*Potyviridae*	9644	97,544	2
	Plantago asiatica mosaic virus	*Alphaflexiviridae*	6128	266,600	4
	Milk vetch dwarf virus segment DNA-R	*Nanoviridae*	1001	87	3
	Milk vetch dwarf virus segment DNA-U1	*Nanoviridae*	989	8	1
	Milk vetch dwarf virus segment DNA-N	*Nanoviridae*	977	42	1
	Milk vetch dwarf virus segment DNA-U2	*Nanoviridae*	981	39	1
	Milk vetch dwarf virus segment DNA M	*Nanoviridae*	985	39	4
	Milk vetch dwarf virus segment DNA-S	*Nanoviridae*	997	218	2
	Milk vetch dwarf virus segment DNA-C	*Nanoviridae*	990	422	1
	Milk vetch dwarf virus segment DNA-U4	*Nanoviridae*	991	143	2
LFLS	Lily mottle virus	*Potyviridae*	9644	43,582	1
	Plantago asiatica mosaic virus	*Alphaflexiviridae*	6128	30	4
LFLY	Lily mottle virus	*Potyviridae*	9644	779	5
	Plantago asiatica mosaic virus	*Alphaflexiviridae*	6128	2642	3
	Lily virus B RNA1	*Secoviridae*	7165	1,139,391	1
	Lily virus B RNA2	*Secoviridae*	3388	2,100,352	2

**Table 3:** Summary of viruses identified from 48 lily hybrid transcriptomes.

Virus Name	Abbreviation	Family	No. of Viral Contigs
Lily virus A	LVA	*Potyviridae*	78
Cycas necrotic stunt virus RNA 1	CNSV-RNA1	*Secoviridae*	1644
Cycas necrotic stunt virus RNA 2	CNSV-RNA2	*Secoviridae*	728
Plantago asiatica mosaic virus	PlAMV	*Alphaflexiviridae*	50
Lily symptomless virus	LSV	*Betaflexiviridae*	478
Lily mottle virus	LMoV	*Potyviridae*	131
Strawberry latent ringspot virus RNA1	SLRSV-RNA1	*Secoviridae*	4
Lily amalgavirus 1	LAV1	*Amalgaviridae*	1
Lily amalgavirus 2	LAV2	*Amalgaviridae*	11

## Data Availability

The data presented in this study are openly available in the National Center for Biotechnology Information (NCBI)’s sequence read archive (SRA) database with respective accession numbers under BioProject accession number PRJNA1313793.

## Supplementary Materials

The following supporting information can be downloaded at: https://www.mdpi.com/article/10.3390/ijms26199598/s1.

## Abbreviations

The following abbreviations are used in this manuscript:

HTS	High-Throughput Sequencing
RT-PCR	Reverse Transcription Polymerase Chain Reaction
LSV	Lily symptomless virus
LMoV	Lily mottle virus
CMV	Cucumber mosaic virus
PlAMV	Plantago asiatica mosaic virus
